# Preoperative CT Angiography Informs Instrumentation in Anterior Spine Surgery for Idiopathic Scoliosis

**DOI:** 10.5435/JAAOSGlobal-D-19-00123

**Published:** 2020-04-01

**Authors:** Alexander A. Theologis, Joel Ramirez, Mohammad Diab

**Affiliations:** From the Department of Orthopaedic Surgery, University of California, San Francisco (UCSF), San Francisco, CA.

## Abstract

The objective of this study is to evaluate whether the artery of Adamkiewicz localization with preoperative CT angiography influences anterior spinal instrumentation.

**Methods:**

Children with idiopathic scoliosis who underwent anterior instrumentation and with a preoperative CT angiography were evaluated retrospectively. Data included curve type, artery of Adamkiewicz level/laterality, surgical approach laterality, number of instrumented levels and segmental vessels ligated, intraoperative neuromonitoring changes, and postoperative neural complications.

**Results:**

Thirty-nine girls and eight boys (mean age 12 years [6.7 to 16.8 years]) were analyzed. Instrumented curves indicate 28 thoracic, 14 thoracolumbar, and seven double major. The artery of Adamkiewicz: T6 (left-1), T8 (left-1), T9 (left-4/right-2), T10 (left-11/right-4), T11 (left-4/right-4), T12 (left-1/right-2), L1 (left-2/right-1), and L2 (left-3/right-2). Four had bilateral dominant segmentals, whereas in nine patients, none was identified. T10 (32%) and left side (57%) were most frequent. On average, 7.1 (4 to 11) segmentals were ligated *per* case (total 355). Dominant vessels were ipsilateral to/within instrumentation levels in 30%.

**Discussion:**

In children with idiopathic scoliosis who underwent anterior instrumentation, the artery of Adamkiewicz was identified on the left in >50% and at T10 in 32%. In one-third of the patients, the artery was within intended surgical levels and resulted in instrumentation modification.

Anterior instrumentation of the scoliotic spine was introduced by Dwyer in 1969.^1^ Several reports have found that segmental vessel ligation at multiple levels in preparation for anterior spine instrumentation is associated with a low neural risk.^2-4^ This may be explained by the fact that segmental vessels at the intervertebral foramen ramify to form an anastomotic network that may compensate for ligation. However, neural risk rises with the number of segmental vessels ligated^5,6,7,8^ because collateral flow becomes more remote and thereby insufficient to reach the level of ligation. A subset of neural complications after anterior instrumentation surgery results from vascular insult. The presumed mechanism is the overwhelming of collateral flow after interruption of flow through a dominant *arteria radicularis anterior magna* (of Adamkiewicz). Onset of injury may be immediate (intraoperative) or delayed (in the postoperative period).^4,9^ Although the incidence is low, the injury is catastrophic because the effect is broad and recovery is poor. CT angiography (CTA) allows preoperative identification and surgical preservation of the artery of Adamkiewicz, thereby increasing the safety of anterior spine instrumentation surgery. We reviewed how CTA informs instrumentation in children who underwent anterior spine surgery for idiopathic scoliosis.

## Methods

After obtaining Institutional Review Board approval, charts were reviewed to identify children with idiopathic scoliosis who had undergone anterior spine instrumentation surgery between October 2012 and June 2017 at a single institution by a single surgeon. Patients were included for analysis based on the presence of a preoperative CTA, which was obtained as a standard safety measure to assess location of the artery of Adamkiewicz for surgical planning.

Clinical data included patient age, sex, and perioperative neural function. Surgical data included the side of surgical approach, number of instrumented levels and segmental vessels ligated, and intraoperative neuromonitoring signals. Radiographic data included curve location, direction of curve apex, and vertebral level of the artery of Adamkiewicz. A minimum follow-up was set at 6 weeks, based on the latest delayed presentation of ischaemic paralysis after aortic surgery reported at 1 month.^10^

**Figure 1:**
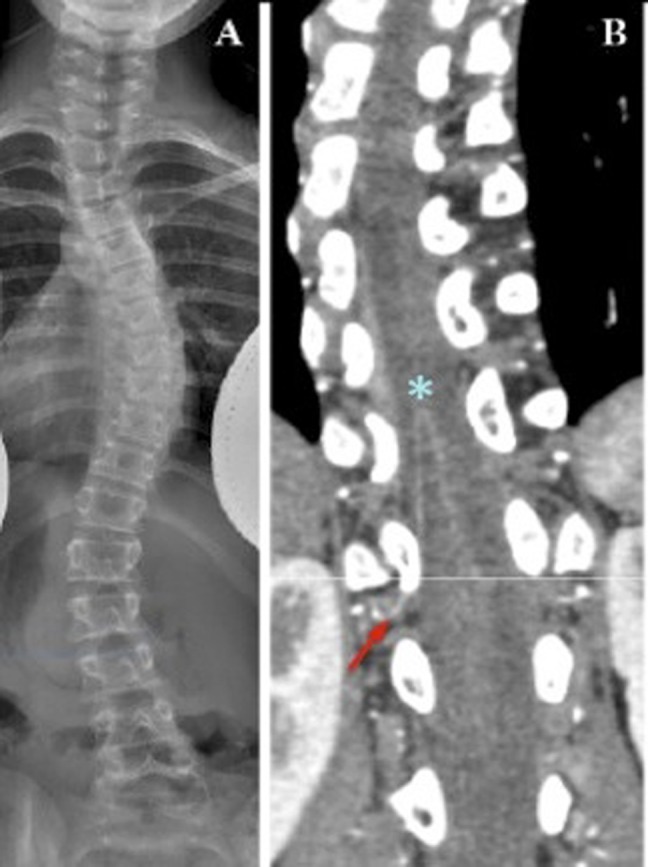
Radiograph showing a 10-year-old premenarcheal girl with progressive thoracic more than lumbar scoliosis despite bracing treatment (**A**) was scheduled to undergo selective convex T5-12 anterior spine instrumentation surgery. A preoperative CT angiography (**B**) demonstrated a dominant artery emerging from the right T12 segmental (red arrow) and continuing to the anterior spinal artery in a “hairpin loop” (blue asterisk). Instrumentation was modified to avoid the dominant artery at the distal-most planned level.

The artery of Adamkiewicz arises from the aorta, courses centrally along the lateral surface of the vertebral body, and passes through the foramen between that vertebra and the one below. Once within the spinal canal, it turns retrograde to feed into the anterior spinal artery 1 to 2 levels craniad because of a mismatch in longitudinal growth between spinal cord and vertebral column.^11^ The artery of Adamkiewicz and the anterior spinal artery resemble the limbs of a hairpin, which are connected at an acute angle. This “hairpin loop” is a conserved and consistent anatomic feature that allows high sensitivity and specificity of identification. All CTAs were read by a fellowship-trained neuroradiologist (Figure 1).

**Figure 2:**
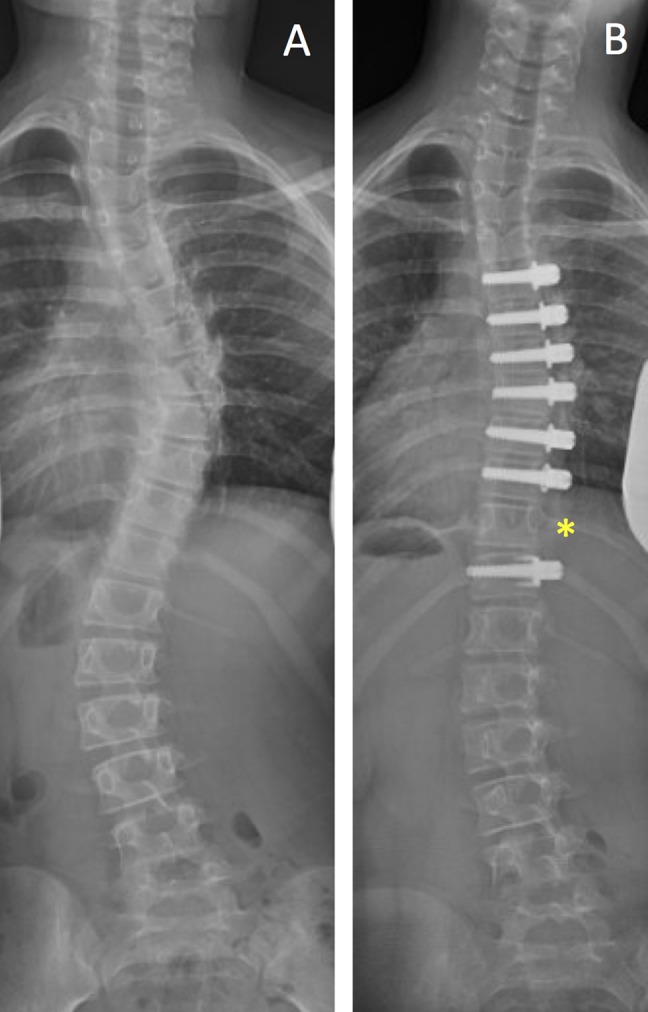
Radiograph showing a 9-year-old premenarcheal girl with progressive thoracic more than lumbar scoliosis who (**A**) underwent a right T5-T11 anterior spine instrumentation. No implant was placed at T11 (asterix) because a preoperative CT angiogram demonstrated the artery of Adamkiewicz at the right T11. In this patient, a second artery of Adamkiewicz was found on the left at L2. There were no neuromonitoring changes intraoperatively and no neural deficits postoperatively.

In the anterior surgical treatment of scoliosis, the spine is instrumented on the convexity of the curve, due in part to easier access (ie, the convexity moves closer to the body wall) and in part to the mechanical advantage of pushing against a deformed spine in comparison with pulling it. The most common implant is a screw inserted into the center of the vertebral body and backed up with a washer to augment fixation. The segmental vessels are sacrificed to provide enough room for adequate implant purchase without injury to the adjacent end plate and intervertebral disk. Before ligation, segmental vessels were reversibly occluded with a Fogarty spring clip for at least 20 minutes to make sure that motor-evoked and sensory-evoked potentials did not change.^12,13^ End vertebrae (by Cobb angle) determine the proximal and distal limits of surgery. A vertebral body where the artery of Adamkiewicz was located on CTA was omitted from instrumentation (Figures 1 and 2), except if the artery was located at either end of the construct; in such cases, fixation was achieved by placing an eccentric screw, which limits compression and thereby correction, or a nonscrew implant such as a staple, which can capture the end vertebra without encroaching on the segmental vessel.

## Results

Forty-seven children met the inclusion criteria—39 girls and 8 boys. The mean age was 12 years (range, 6.7 to 16.8 years). Patient characteristics, curve types, and surgical details are presented in the Supplemental Table, http://links.lww.com/JG9/A68. All curve instrumentations were primary procedures. Two patients underwent a second primary procedure for progression of an originally spared, uninstrumented thoracic curve that progressed after lumbar spine instrumentation because of continued growth and thereby progression of a previously small scoliosis (<40°) that did not meet the criteria for surgical treatment at index procedure (numbers 3 and 19).

Curves instrumented were 28 right thoracic, 13 left and 1 right thoracolumbar, and seven right thoracic-left lumbar (double major, Supplemental Table, http://links.lww.com/JG9/A68). The levels of the artery of Adamkiewicz included T6 (left-1), T8 (left-1), T9 (left-4, right-2), T10 (left-11, right-4), T11 (left-4, right–4), T12 (left-1, right-2), L1 (left-2, right-1), and L2 (left-3, right-2, Table 1). T10 (32%) and left side (57%) were most frequent. Four patients (8.5%) had bilateral dominant feeder vessels to the anterior spinal artery, whereas in nine patients (19%), no dominant feeder was identified. On average, 7.1^4,5,6,7,8,9,10,11^ segmental vessels were ligated *per* case, for a total of 355. The artery of Adamkiewicz was ipsilateral to and within the levels of instrumentation in 14 patients (30%), in whom the vessel was not sacrificed and instrumentation was modified to accommodate. Modification of instrumentation included capture of an end vertebra with a staple (Figure 1) and omission of a level within the construct (Figure 2). There were no intraoperative neuromonitoring changes and no postoperative neural complications. There were no acute or delayed adverse events associated with the CTAs.

**Table 1:** Vertebral Level at Which the Artery of Adamkiewicz was Visualized by CT Angiography

Level	Left	Right
T6	1	0
T8	1	0
T9	4	2
T10	11	4
T11	4	4
T12	1	2
L1	2	1
L2	3	2

More than one half of the dominant feeders entered from the left side of the spine. One third coursed over the T10 vertebral body. Nine patients had no identifiable dominant artery.

## Discussion

Blood supply to the thoracic and lumbar regions of the spinal cord by the anterior median longitudinal artery is incomplete without augmentation by segmental feeders from the aorta. The largest of these feeders is the *arteria radicularis anterior magna*, which also is known as the artery of Adamkiewicz. At the intervertebral foramen, each segmental vessel ramifies to contribute to an anastomotic network that may compensate for individual ligation. The location of the artery of Adamkiewicz has clinical relevance when segmental vessels are ligated, both to the vascular surgeon operating on the aorta and to the spine surgeon instrumenting the vertebral bodies. Disruption of the anterior vasculature can lead to spinal cord injury.^9,14-16^ Risk increases with the number of segmentals ligated^5,6,7,8^ because collateral flow has to travel further to reach the site of ligation.

In this study, the artery of Adamkiewicz was identified on the left side of the spine in more than one half and at T10 in one-third of cases; these findings are consistent with other literature,^17,18^ identifying the left low thoracic segment as a critical vascular zone of the spinal cord.^19^ Variability in the anatomy of the artery of Adamkiewicz is reflected in the reports of right side entry and a range of levels from T6 to L5.^3,17^ In our series, the artery of Adamkiewicz was identified on the right in 43% of children and ranged from T6 (1 case) to L2 (4 cases). In addition, we found that the artery of Adamkiewicz to be bilateral in 8.5% of patients, which is consistent with the notion that there may be more than one critical feeder.^3,17,20,21^

The actual risk of segmental vessel sacrifice remains unclear. A retrospective report of approximately 6,000 segmental vessel ligations in 1,197 consecutive anterior procedures and another of 2,651 in 173 patients, respectively, noted no neural deficits in patients undergoing anterior spinal instrumentation with fusion^2,3^. Furthermore, selective ligation of the artery of Adamkiewicz did not produce paraplegia in a study of rhesus monkeys.^22^ Neither the laterality of segmental ligation nor the specific vessel per se is critical alone. In the studies cited above, no patient underwent more than a five-level operation because the anterior approach was intended to limit fusion levels. When more levels have to be included to address a curve from end vertebra to end vertebra, or when more than one curve must be addressed, the number of vessels ligated may increase beyond the ability for collateral flow to compensate, thereby reducing total volume of blood flow and risking injury to the spinal cord.

A dominant segmental vessel was identified in 81% of the patients, suggesting that CTA is reliable.^23,24,25,26^ We used CTA to reduce variability and error. CTA is relatively cheap, readily available, and quick. The last feature is of particular importance in young children, who may require sedation to avoid movement artifact for prolonged imaging. CTA has the added benefit of less distortion in the presence of implants, as in evaluation before revision. Reported adverse events associated with CTA include contrast extravasation producing a local inflammatory reaction (0.1% to 0.9%), allergy that may be managed by anti-inflammatory medication (0.04% to 14%), and contrast-induced nephropathy (1% to 2%).^27,28^ No adverse events attributable to the CTAs occurred in our patients. CTA delivers 3 mSv, of relevance because radiation exposure to children should be minimized on account of malignancy risk.^29-31^ Although this amount of radiation is higher than the maximum allowed equivalent dose of radiation to members of the general public *per annum* (1 mSv/yr), the average American exposure to ionizing radiation is 3 mSv/yr.^32^ The risk of malignancy from one CT is unknown^33^*.* Magnetic resonance angiography has recently been shown to be an effective alternative to CTA for the study of spinal cord perfusion.^11,23,24,25,26,34,35^ The avoidance of radiation exposure must be balanced against the requirement of anesthesia for sedation of a young child undergoing an MRI of the spine and the reduced availability of this imaging modality.

Anterior instrumentation of the spine without sacrifice of segmental vessels has been described in adults.^36^ Vertebral staples are inserted across the end plates and intervertebral discs and as such do not disturb the segmental vessels. There are several considerations, however, that preclude segmental sparing when using a screw as anchor. First, the vertebral body may be too small (eg, in a child) or too osteopenic to allow mobilization of the segmental vessels and off-center screw instrumentation that provides sufficiently stable fixation. Second, as a screw diverges form the center of a vertebral body, it approaches and risks the end plate, which in turn may injure the adjacent intervertebral disk. Avoidance of the segmental vessels requires that the screw head (which is wider than the screw shaft) be kept proud off the vertebral body surface, which may impinge against adjacent soft-tissue structures such as the great vessels (aorta and vena cava). Even if a screw may be placed eccentrically in the vertebral body to avoid the segmental vessels, the screw is typically a backup by a staple or washer to improve fixation; these increase the footprint of the screw and would encroach on the segmentals. The exclusion of a staple or washer would weaken the construct, thereby reducing the ability to correct the curve and the stability of the spine.

We temporarily clamp segmental vessels at each level for 20 to 30 minutes to simulate ligation and to allow sufficient time for detection of changes in motor-evoked and sensory-evoked potentials.^12,37,38^ Although we did not detect any intraoperative or postoperative neural deficits, delayed ischaemia and spinal cord injury is possible^40^. In a pig model, recruitment of collateral flow from ipsilateral remote and contralateral segmental vessels requires up to 96 hours to reach >90% preligation perfusion.^39^ Hypotension, continued haemorrhage, and/or haemodilution after operation may undermine compensatory mechanisms to the point of ischaemia, leading to late-onset spinal cord injury. Supporting the experimental model is the latest report of paralysis after scoliosis correction in a child at 72 hours.^40^ In our experience before CTA, a 15-year-old girl had normal neural signals at conclusion of anterior thoracolumbar spinal fusion with instrumentation (T11-L3; left approach) and a normal physical examination in the postsurgical care unit, only to develop paraplegia 1 day after operation. Postoperative MRI was consistent with a spinal cord infarction just proximal to the surgical site. This potential for delayed injury—undetectable during operation—lends support to the omission of the artery of Adamkiewicz as identified on preoperative CTA and the incompleteness of temporary vessel clamping.^36^

Our study has limitations. It is a retrospective evaluation of a preliminary cohort. Surgical variability because of heterogeneity in curve characteristics and surgical details (ie, duration, number of levels instrumented, and number of segmentals ligated) was minimized by including cases from a single surgeon. Absence of a comparative group decreases this study's level of evidence. However, a retrospective cohort analysis that includes children without a preoperative CTA would neither be informative nor accurate because the level and laterality of the artery of Adamkiewicz would not be known. Although a prospective cohort analysis would be ideal, deliberate sacrifice of the artery of Adamkiewicz to test its importance would not be possible.

## Conclusions

CTA helps to elucidate the anatomy of the artery of Adamkiewicz. In children with idiopathic scoliosis who underwent anterior spine instrumentation surgery, the artery of Adamkiewicz was identified on the left in more than one half and at T10 in one-third. Because the artery was within the intended surgical levels in one-third of the cases, CTA allowed modification of instrumentation to avoid ligation of the artery of Adamkiewicz. Preoperative CTA may be of benefit in patients who undergo anterior spine instrumentation surgery for idiopathic scoliosis, although this should be confirmed with larger cohorts in prospective and comparative analyses.

## Supplementary Material

SUPPLEMENTARY MATERIAL
