# Supplementary material for: Preoperative CT Angiography Informs Instrumentation in Anterior Spine Surgery for Idiopathic Scoliosis
Source: J Am Acad Orthop Surg Glob Res Rev. 2020 Apr 1;4(4):e19.00123. doi: 10.5435/JAAOSGlobal-D-19-00123 (PMC7188266; doi:10.5435/JAAOSGlobal-D-19-00123)
Supplement: SUPPLEMENTARY MATERIAL [file jg9-4-e19.00123-s001.docx]

| **#** | **Gender** | **Age**  **(yr)** | **Curve** | **Tether** | **Dominant**  **Vessel**  **(level)** | **Dominant**  **vessel**  **(laterality)** | **Approach (laterality)** | **Instrumented**  **Level** | **Skipped**  **Level** |
| --- | --- | --- | --- | --- | --- | --- | --- | --- | --- |
| **1** | Female | 13 | Thoraco-  Lumbar | Screw-cord | Invisible | Not  visualized | Left | T10-L3 | N/A |
| **2** | Female | 12 | Thoracic | Screw-cord | T10 | Right | Right | T6-L1 | T10 (right) |
| **3** | Female | 9 | Thoraco-lumbar | Screw-cord | T11 | Right | Left | T11-L3 | N/A |
|  |  | 10 | Thoracic | Screw-cord | T11 | Right | Right | T5-11 | T11 (right) |
| **4** | Female | 10 | Thoracic | Screw-cord | L2 | Left | Right | T5-T12 | N/A |
| **5** | Female | 11 | Thoracic | Screw-cord | T10 | Left | Right | T6-T12 | N/A |
| **6** | Female | 12 | Thoracic | Screw-cord | T10 | Left | Right | T6-L1 | N/A |
| **7** | Female | 11 | Thoracic | Screw-cord | T6 & T9 | Left & Right | Right | T6-T12 | T9 (right) |
| **8** | Male | 13 | Thoraco-lumbar | Screw-cord | T10 | Left | Right | T11-L3 | N/A |
| **9** | Female | 10 | Thoracic  & lumbar | Screw-cord | T10 | Left | Right & Left | T5-T11 (Right)  T12-L3 (Left) | N/A |
| **10** | Male | 13 | Thoracic | Screw-cord | T10 | Left | Right | T4-T12 | N/A |
| **11** | Male | 11 | Thoraco-lumbar | Screw-cord | T10 | Left | Left | T11-L3 | N/A |
| **12** | Female | 9 | Thoracic | Screw-cord | L2 & T11 | Left & Right | Right | T5-T12 | T11 (right) |
| **13** | Female | 7 | Thoracic | Stapling | T11 | Left | Right | T5-12 | N/A |
| **14** | Female | 11 | Thoracic | Screw-cord | T12 | Right | Right | T5-12 | T12 (right) |
| **15** | Female | 7 | Thoracic  & Lumbar | Screw-cord | T10 | Right | Right & Left | T5-11 (Right)  T11-L3 (Left) | T10 (right) |
| **16** | Female | 11 | Thoracic | Screw-cord | T10 | Right | Right | T5-12 | T10 (right) |
| **17** | Female | 13 | Thoracic  & Lumbar | Screw-cord | T11 & L2 | Right & Left | Right & Left | T5-11 (Right)  T11-L3 (Left) | L2 (left) |
| **18** | Female | 11 | Thoracic | Screw-cord | T12 | Left | Right | T5-12 | N/A |
| **19** | Male | 11 | Lumbar | Screw-cord | T10 | Left | Left | T11-3 | N/A |
|  |  | 13 | Thoracic | Screw-cord | T10 | Left | Right | T5-10 | N/A |
| **20** | Female | 12 | Thoracic | Screw-cord | T10 | Left | Right | T6-12 | N/A |
| **21** | Female | 13 | Thoracic | Screw-cord | T9 | Left | Right | T5-12 | N/A |
| **22** | Female | 7 | Thoracic | Screw-cord | T9 | Right | Right | T5-12 | T9 (right) |
| **23** | Female | 13 | Thoracic | Screw-cord | Invisible | Not  visualized | Right | T5-12 | N/A |
| **24** | Female | 12 | Thoraco-lumbar (thoracic) | Screw-cord (PSF) | T8 | Left | Left | T11-L3 | None |
| **25** | Female | 10 | Thoracic  & Lumbar | Screw-cord | T10 | Left | Right &  Left | T5-11 (Right)  T11-L3 (Left) | None |
| **26** | Female | 12 | Thoracic | Screw-cord | Invisible | Not  visualized | Right | T5-12 | None |
| **27** | Female | 14 | Thoracic | Screw-cord | Invisible | Not  visualized | Right | T5-12 | None |
| **28** | Female | 13 | Thoracic | Screw-cord | T11 | Left | Right | T6-12 | None |
| **#** | **Gender** | **Age**  **(yr)** | **Curve** | **Tether** | **Dominant**  **Vessel**  **(level)** | **Dominant**  **vessel**  **(laterality)** | **Approach (laterality)** | **Instrumented**  **Level** | **Skipped**  **Level** |
| **29** | Female | 15 | Thoraco-lumbar (thoracic) | Screw-cord (PSF) | Invisible | Not  visualized | Left | T12-L3 | None |
| **30** | Female | 12 | Thoracic | Screw-cord | T9 | Left | Right | T5-12 | None |
| **31** | Female | 13 | Thoraco-lumbar | Screw-cord | L2 | Right | Left | T11-L3 | None |
| **32** | Male | 16 | Thoracic | Screw-cord | T9 | Left | Right | T6-12 | None |
| **33** | Female | 13 | Thoracic | Screw-cord | Invisible | Not  visualized | Right | T5-11  (T12 convex tether) | T12 (right) |
| **34** | Female | 16 | Thoraco-lumbar (thoracic) | Screw-cord (PSF) | T10 | Left | Left | T12-L3 | None |
| **35** | Female | 11 | Thoraco-lumbar (thoracic) | Screw-cord (PSF) | Invisible | Not  visualized | Left | T12-L4 | None |
| **36** | Female | 11 | Thoracic | Screw-cord | T11 | Left | Right | T6-L1 | None |
| **37** | Female | 12 | Thoraco-lumbar | Screw-cord | L2 | Right | Left | T11-L3 | None |
| **38** | Female | 16 | Thoraco-lumbar | Screw-cord | T9 | Left | Left | T12-L4 | None |
| **39** | Female | 13 | Thoraco-lumbar | Screw-cord | T11 | Right | Left | T11-L3 | None |
|  |  |  | Thoracic | Screw-cord | T11 | Right | Right | T4-11 | T11 (right) |
| **40** | Male | 15 | Thoracic | Screw-cord | T10 | Right | Right | T5-12 | T10 (right) |
| **41** | Female | 13 | Thoracic  & Lumbar | Screw-cord | Invisible | Not  visualized | Right &  Left | T5-10 (Right)  T11-L3 (Left) | None |
| **42** | Female | 8 | Thoracic (thoraco-lumbar) | Screw-cord (PSF) | T11 | Right | Right | T5-11 | T11 (right) |
| **43** | Female | 13 | Thoraco-lumbar | Screw-cord | L1 | Left | Left | T10-L3 | L1 (left) |
| **44** | Male | 12 | Thoracic | Screw-cord | T11 | Left | Right | T5-L1 | None |
| **45** | Female | 14 | Thoracic | Screw-cord | Invisible | Not  visualized | Right | T4-12 | None |
| **46** | Female | 17 | Thoraco-lumbar | Screw-cord | Invisible | Not  visualized | Left | T11-L3 | None |
| **47** | Female | 10 | Thoracic (thoraco-lumbar) | Screw-cord (PSF) | L1 | Left and Right | Left | T11-L3 | None |

**Table 1.** Patients and anatomic characteristics

One patient (13) underwent a stapling procedure. One patient (16) underwent a primary stapling procedure followed by a revision screw-cord procedure for progression. Two patients (3,19) underwent a sequential procedure for interval progression of an originally spared curve. Instrumentation was modified to avoid an artery of Adamkiewicz in one third of cases.
